# Commissioning of a near real‐time in vivo film dosimetry system

**DOI:** 10.1002/acm2.70525

**Published:** 2026-02-25

**Authors:** Yunjie Yang, Seng Boh Lim, Indra Das, Jeonghoon Park, Grace Tang, Dongxu Wang, Megan Hyun, Xiuxiu He, Maria Chan

**Affiliations:** ^1^ Department of Medical Physics Memorial Sloan Kettering Cancer Center New York New York USA; ^2^ Department of Radiation Oncology Northwestern University Feinberg School of Medicine Chicago Illinois USA

**Keywords:** in vivo dosimetry, radiochromic film, radiotherapy, skin dose measurement

## Abstract

**Background:**

Accurate in vivo dosimetry is crucial for dose monitoring of cardiac implantable electronic devices (CIED) and for dose verification for special procedures such as total body irradiation (TBI) and total skin electron therapy (TSET).

**Purpose:**

A new near real‐time in vivo dosimetry system using radiochromic films (RCF) is investigated for clinical use in megavoltage external beam radiotherapy.

**Methods:**

The Pnt‐Dos™ in vivo dosimetry system comprises of a new type of RCF and a dedicated software module. Each Pnt‐Dos device is a small piece of RCF individually packed with a unique QR code for identification and record keeping. Different from the traditional film dosimetry workflow, where a film developing time of at least 16 hours is recommended, a near real‐time dose readout can be achieved with the Pnt‐Dos system using a novel calibration procedure. This involves an automated scanning process at user‐specified time intervals, utilizing auto‐region of interest (ROI) detection and triple‐channel calibration to capture the time‐resolved post‐irradiation growth. Two standard Epson scanner models (V600/13000XL) were used to cross‐validate readouts and accommodate users who may prefer to utilize existing 13000XL scanners rather than acquire an additional V600 for in vivo dosimetry. The dosimetric accuracy was evaluated over a range of 15–400 cGy. Angular dependence was studied in 45° increments over 360°, normalized to the response at 0°, at 250 cGy using a cylindrical phantom. Energy dependence was evaluated for four photon energies (6 MV, 6 MV FFF, 10 MV FFF, 15 MV) and five electron energies (6 MeV, 9 MeV, 12 MeV, 16 MeV, and 20 MeV). Long‐term reproducibility/stability were assessed with nine devices with different doses under identical conditions, alongside daily scans of quality control (QC) devices over three months.

**Results:**

The system provides accurate dose measurements across high‐ and low‐dose ranges. All readings were within specification: accuracy was < ± 5 cGy for doses ≤ 80 cGy doses (max discrepancy 6.0 cGy), and < ± 5% for doses > 80 cGy on average (max discrepancy 5.1%). Angular dependence showed a maximum variation of 2.6% ± 2.1% when the beam passed through the posterior oblique side of the device. Daily QC/reproducibility tests confirmed system constancy of 0.1% average day‐to‐day variation. Energy dependence analysis revealed deviations of up to 4.9% ± 2.3% for all photon and electron energies compared to 6 MV photons, indicating the need for energy correction during commissioning. Film readings were compared with ion chamber measurements at 10 × 10 cm^2^, d_max_, 100 cm SAD (photons) or 100 cm SSD (electrons). Both scanners provided comparable readouts, within 1.3 cGy for doses ≤ 80 cGy and 0.6% for doses > 80 cGy. Based on these findings, user guidelines were established to ensure optimal performance and accuracy.

**Conclusion:**

The new film‐based in vivo dosimetry system provides an automated workflow that enables consistent, time‐independent, and near real‐time readout with a user‐friendly design that simplifies handling and analysis, thereby streamlining in vivo dosimetry measurements. It also provides a traceable record of the patient dosimetry.

## 1 INTRODUCTION

In vivo dosimetry is an integral part of a comprehensive quality assurance (QA) program in modern radiation therapy.^1^, ^2^ It aims to directly measure the radiation dose administered to the patient during treatment. This goal poses specific requirements for the detection system compared to other types of in vitro or in phantom radiation dosimetry measurements. Numerous detector types have been explored and utilized for this purpose such as thermoluminescence detectors (TLD), optically stimulated luminescence detectors (OSLD), diodes, scintillators, radiochromic films (RCFs), and electronic portal imaging detectors (EPID).

RCFs are constructed with one or more active layers of monomers in a crystal form arranged in thin two‐dimensional films. The monomers undergo polymerization upon absorbing ionization radiation. The polymerization process in turn changes the light absorbance spectrum of the film, resulting in a color change. The amount of color change can be correlated with the amount of ionizing radiation absorbed, making it an effective dosimeter.

RCF has been widely used in radiotherapy physics dosimetry measurements for various purposes due to its versatility and excellent spatial resolution.^3^, ^4^ It has also been used to perform in vivo or in vitro dosimetry measurements,^5^, ^6^, ^7^, ^8^, ^9^, ^10^, ^11^, ^12^, ^13^, ^14^, ^15^, ^16^, ^17^ including special procedures such as intraoperative procedures,^9^, ^10^ total skin electron treatment (TSET)^11^, ^12^, ^13^ and total body irradiation (TBI).^14^, ^15^, ^16^, ^17^

However, using RCFs for in vivo dosimetry comes with several unique challenges. First, it is often clinically desirable, if not mandatory, to obtain the results of in vivo dosimetry measurements as soon as possible, as this information will be used for clinical decision‐making before the patient receives the next treatment fraction. The post‐irradiation optical density (OD) growth is a characteristic feature of RCFs; a common practice to account for the different elapsed times since irradiation between the calibration and measurement films is to wait a sufficiently long time (e.g., 16 to 24 hours as recommended by AAPM TG‐235^3^) before reading the measurement films to ensure dosimetric accuracy. Lewis *et al* developed an efficient calibration and scanning process for film dosimetry, known as the “one‐scan” protocol, providing a solution to read the measurement films as soon as a few minutes post irradiation.^18^ However, an additional exposure of a RCF with a known reference dose immediately before or after the in vivo dosimetry measurement is needed, introducing additional requirements on the resources and the clinical workflow.

Another challenge of using RCFs for in vivo dosimetry is the wide dose range incurred in measurements per various clinical needs. For example, low doses of a few cGy are expected in out‐of‐field measurements to ensure the safe operations of cardiac implantable electronic devices (CIEDs); higher doses on the order of hundreds of cGy can be observed during in‐field dose verification measurements for skin treatments using electrons. This requires the RCFs to be sensitive and accurate across a wide range of dose levels. In contrast, previous generations of commercially available RCFs for radiotherapy are not typically designed to achieve high sensitivity and accuracy in the very low doses of a few cGy—the manufacturer's optimal dose range specification for EBT3 and EBT4 RCFs starts at 20 cGy.^19^

Recently, Ashland Inc. (Bridgewater, NJ, US) introduced an FDA‐cleared RCF‐based in vivo dosimetry system called “Pnt‐Dos™”.^20^ The system consists of a new type of RCF, designed to be sensitive to both low and high doses. Additionally, the in vivo dosimetry analysis module, which is integrated within the FilmQA Pro v8 (Ashland Inc., Bridgewater, NJ, US), has features that are specifically designed and streamlined to meet the requirements of in vivo dosimetry. It allows the user to build a time‐resolved calibration set, which enables reading the in vivo measurement device within minutes after irradiation by using the corresponding calibration curve built at a similar time post‐irradiation, without the need to perform an additional irradiation as is done in the “one‐scan” protocol.^18^ In this study, the commissioning and our initial clinical experience with the Pnt‐Dos system is presented.

## 2 METHODS AND MATERIALS

### 2.1 Pnt‐Dos system

#### 2.1.1 Pnt‐Dos device

**FIGURE 1:**
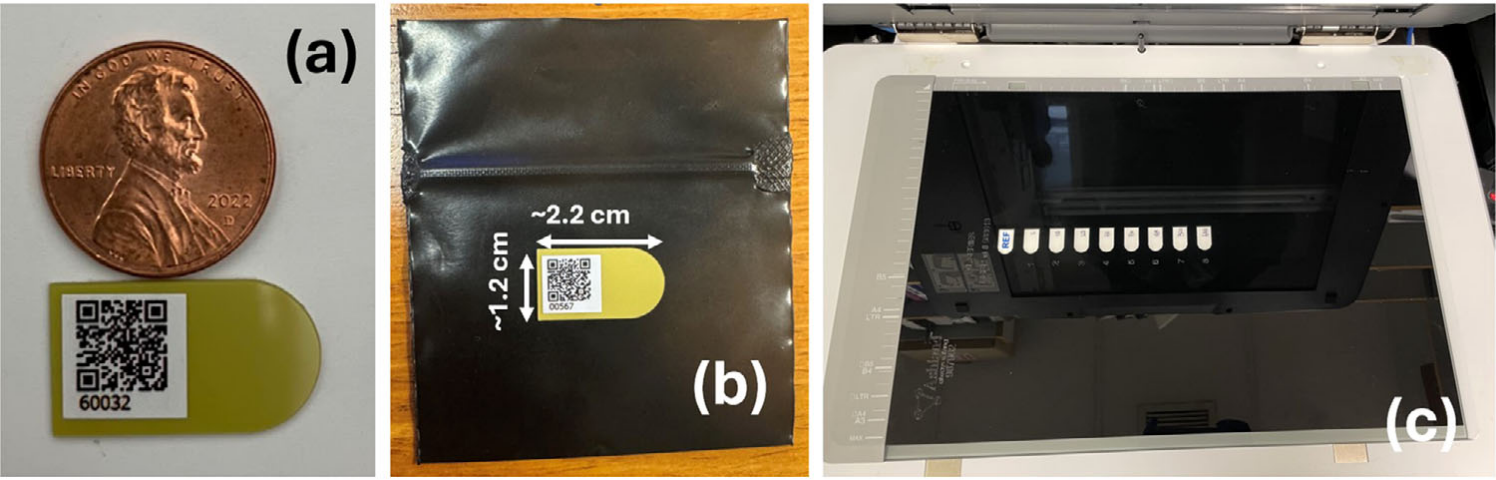
(a) A Pnt‐Dos device compared to a penny. (b) and on top of its protective UVC‐protection pouch with the approximate dimensions. (c) The 9‐slot device holder template for the Epson XL series scanner with Pnt‐Dos devices.

The Pnt‐Dos system consists of two main components, the Pnt‐Dos device and the in vivo dosimetry software module. The Pnt‐Dos device, shown in Figure 1, comprises a newly designed RCF on an opaque substrate. Each device is labelled with a QR code and is individually packed in an ultraviolet C (UVC)‐protected pouch. For record keeping, the QR code is read out during the scanning process and contains information about the device such as its serial number, manufacturing production lot, and the expiration date. The vendor specifies a 4‐month shelf life for each batch; however, functionality may be maintained beyond six months with monthly calibration and validation post‐expiration or pre‐use (see “re‐commissioning steps” in the Discussion section). The device can be attached to the patient's skin using the sticker on its back after peeling off the white layer. To ease scanning workflow, the Pnt‐Dos in vivo dosimetry system provides a scanner‐specific device holder, as shown in Figure 1(c), to facilitate easy and consistent placement of the Pnt‐Dos devices on the scanner bed. The holder has nine predefined slots for Pnt‐Dos device placement and there are two variations of the device holder: a smaller one to fit the Epson V600 and a larger one for the Epson XL series scanners. The manufacturer requires the Pnt‐Dos devices to be stored in temperatures between 4°C and 6°C,^21^ which can be provided using commercially available mini‐refrigerators, to achieve the specified dosimetric accuracy.

#### 2.1.2 Pnt‐Dos software module

The in vivo dosimetry module is provided within the FilmQA Pro v8 software. It has three submodules: Scanner Check (QA), Film Calibration, and Pnt‐Dos Reader.

The “Scanner Check (QA)” submodule is an automated routine that assesses the scanner readout consistency via 20 blank scans. A region of interest (ROI) corresponding to the area where the devices will be scanned is analyzed for pixel value consistencies for three color channels (blue, green, and red). The check passes if the variation of all three channels is <3%, defined by the ratio between the maximum and the minimum pixel values among the 20 scans. In our experience, the maximum observed variation was 1.9%.

The “Film Calibration” submodule is the key to enabling the near real‐time readout of the measurement, taken just minutes after irradiation. Similar to other RCFs, a group of Pnt‐Dos devices is irradiated to a range of known doses for calibration purposes. Before initiating the automated scanning routine in this submodule, a preview scan is performed. Based on this preview image, the user verifies and adjusts, if necessary, to ensure that the automatically detected ROIs are in the desired locations. Then, the user specifies the following parameters for the automated calibration: the known doses for each device (including an unirradiated reference device), the time of irradiation of the calibration devices (e.g., the midpoint of the irradiation interval for all calibration devices can be used), and the time intervals at which the automated scanning will be performed. Once these parameters are specified, the automated scanning and calibration routine can be initiated. In this study, the automated scans are performed at every five (5) min for the first four (4) hours and every 15 min after 4 hours for a total of 24 hours. A progress bar displays information to the user, including the number of scans already performed and a countdown timer for the next scan.

**FIGURE 2:**
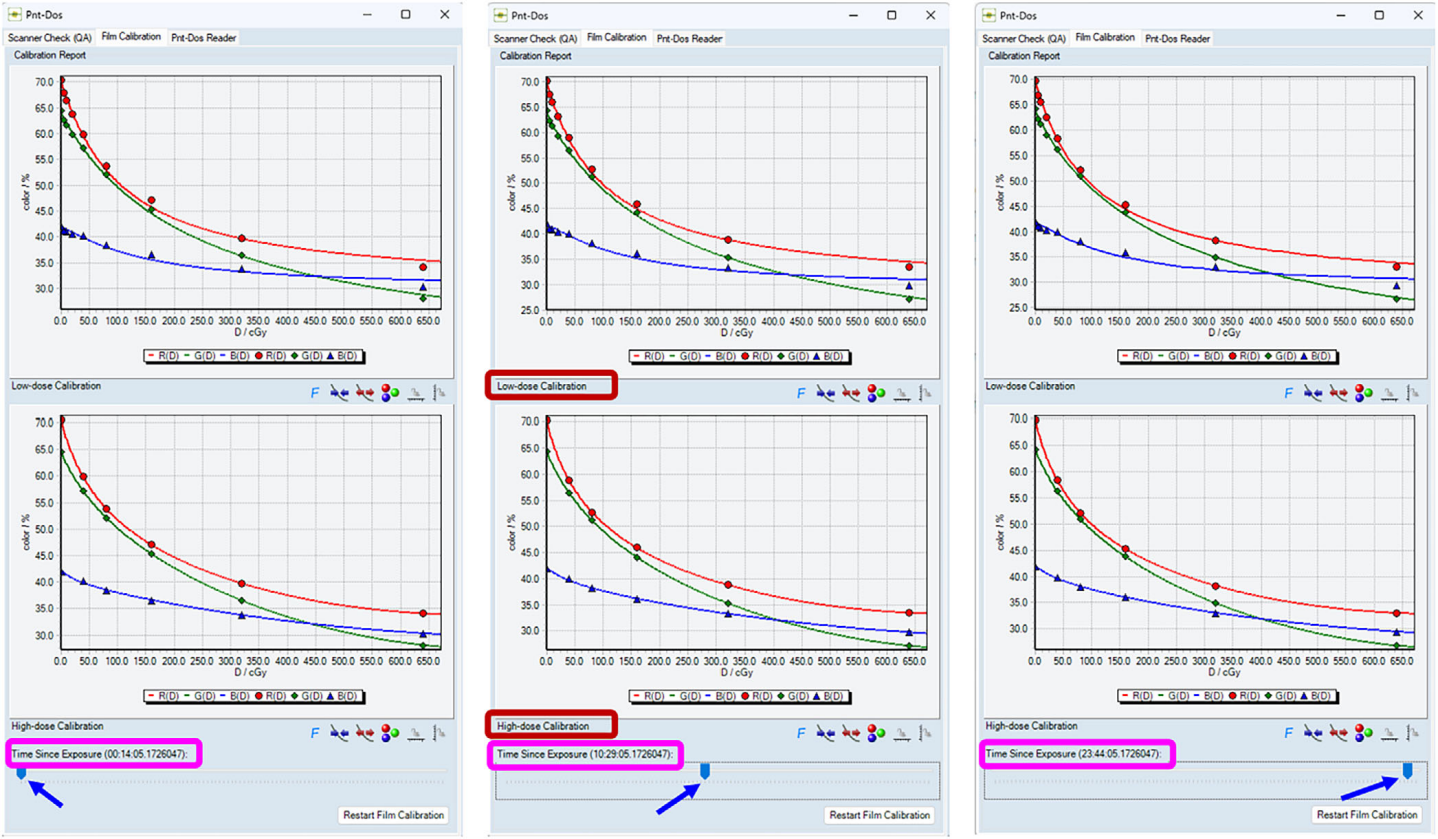
Examples of the automatically generated calibration curves. The curves in the top (bottom) row are the low‐dose (high‐dose) curves, as indicated by the red boxes. The blue arrows indicate the slider bar allowing the users to review all the calibration curves, depending on the time since exposure, in the time‐resolved calibration curve set. Calibration curves from an early (left), intermediate (middle), and a late (right) time points since irradiation are shown. The exact times of scanning since exposure are indicated by the magenta boxes.

After all the scans are finished, the user can review the calibration curves of the pixel value as a function of dose, which are overlaid with the data points, at each time point of the automated scanning routine via a slider bar tool. The result of this calibration routine is a set of calibration curves, characterizing the post‐irradiation OD growth over time. For each scan, a pair of calibration curves is established: a low dose curve and a high dose curve. Both curves are obtained by fitting the dose‐response data of pixel value versus the delivered doses to an analytical function. The low dose curve, for doses below 80 cGy, is fitted to an exponential function, whereas the high dose curve, for doses above 80 cGy, is fitted to a third‐order polynomial. The functional forms of the fitting functions are not modifiable by the user. Figure 2 shows some examples of the calibration curves at three time points post‐irradiation.

The “Pnt‐Dos Reader” submodule performs the dose reading of an irradiated device. Three scans are performed, and the pixel values for the ROIs are averaged. The user needs to input the time of irradiation for the device. From the time of reading, which is automatically extracted from the computer hosting the software, and the user‐entered time of irradiation, the time‐since‐irradiation is computed and used to look up the corresponding calibration curve from the calibration set that is closest to the entered time‐since‐irradiation. This time‐matched calibration curve is used to map pixel values to dose utilizing a triple‐channel dosimetry formalism,^22^ and a dose report can be generated. The software also offers options to save scanned images and pixel values for further analysis. Many helpful items are shown in the dose report, such as the calibration curve and information about the device and scanner. The software facilitates production batch‐dependent calibration by using device barcodes to prevent the application of a calibration curve from an incorrect lot—only the calibration curves from the same batch are displayed and available to use for reading.

### 2.2 Dosimetric evaluation of the Pnt‐dos system

Pnt‐Dos devices from Lot 10242401 and 04182503 were used, including a total of 6 boxes and about 300 Pnt‐Dos devices.

#### 2.2.1 Calibration

The calibration curves were established using nine devices, with one reference device at 0 cGy and eight devices irradiated to 5, 10, 20, 40, 80, 160, 320, and 640 cGy with 6 MV beams in solid water at 1.5 cm depth, 100 cm SAD, following institutional calibration dosimetry protocol. The irradiation monitor units were set according to calibration by ion chamber (Standard Imaging Exradin A12 0.65cc thimble chamber with ADCL calibration) at the same location and depth in the solid water. The output of the Linac was calibrated per AAPM TG‐51 protocol^23^ with 1% uncertainty.

#### 2.2.2 Dose accuracy

For validating dose accuracy, separate sets of Pnt‐Dos devices were irradiated to eight dose levels, different from those used in calibration, using 6 MV beams under identical irradiation conditions: 15, 30, 75, 100, 250, and 400 cGy. There were 15 Pnt‐Dos devices used in each dose group. Dose accuracy was evaluated by comparing the reported dose from the Pnt‐Dos system to the expected delivered dose. After irradiation, the validation devices could be read at any time if a valid calibration curve corresponding to the same time since irradiation, as specified by the user, was available. Multiple readings were taken using the same devices for a range of time‐since‐irradiation between 15 min and almost 24 hours to evaluate the consistency of the dose readings. Users must wait for the first calibration curve; for example, if the first curve is generated 15 min post‐irradiation, readings can start after 15 min. Readout can occur any time thereafter.

#### 2.2.3 Energy dependence

Depending on the elemental composition of the RCF in the Pnt‐Dos device, it may exhibit dependence on the beam energy. A set of Pnt‐Dos devices, two in each energy group, were irradiated to 250 cGy with four photon beam energies: 6 MV, 6 MV flattening filter free (FFF), 10 MV FFF, and 15 MV; and five electron beam energies: 6 MeV, 9 MeV, 12 MeV, 16 MeV, and 20 MeV. The irradiations were performed based on institutional calibration dosimetry protocol in solid water, which was cross calibrated with TG‐51 reference dosimetry results: 100 cm SAD for photons at depths of 1.5 cm for 6 MV and 6 MV FFF, and 3 cm for 10 MV FFF and 15 MV); 100 cm SSD for electrons at depths of 1.5 cm for 6 MeV, 2 cm for 9 MeV, 2.5 cm for 12 MeV, 16 MeV, and 20 MeV. The readings of all other energies were compared to the 6 MV reading, and the energy correction factors were derived after accounting for the output differences between the beams.

#### 2.2.4 Angular dependence

**FIGURE 3:**
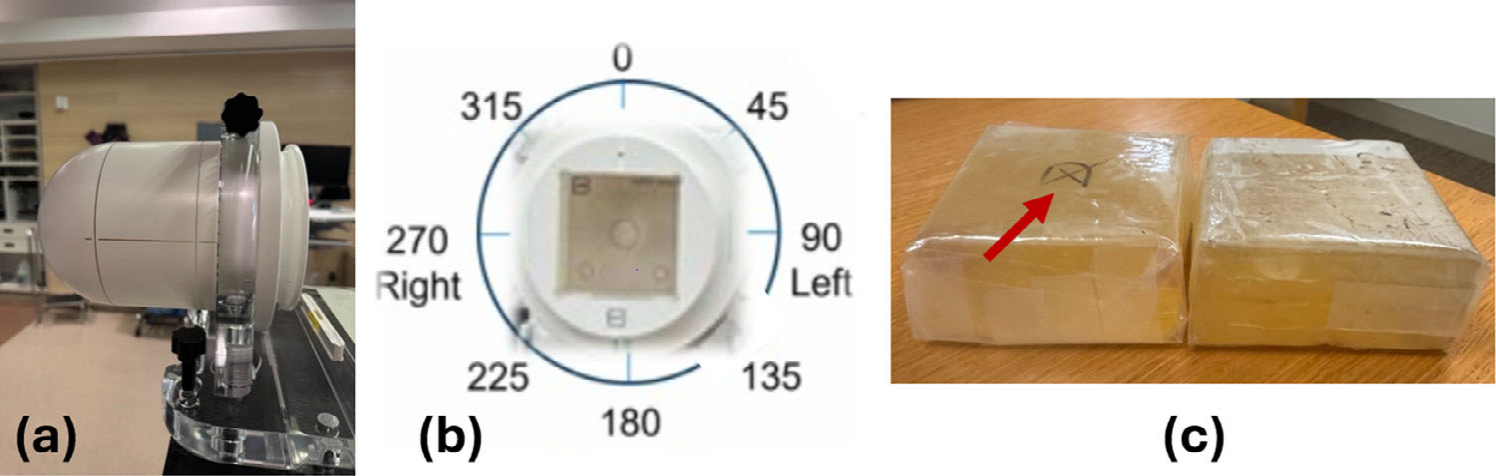
(a) StereoPHAN setup on the TrueBeam couch, (b) axial view of the phantom, and (c) the two halves of the custom‐made bolus insert. The marks indicated by the red arrow show the location of where the Pnt‐Dos was placed.

The Pnt‐Dos device has an opaque substrate with the active RCF component attached. This asymmetric design warrants investigation into its angular dependence. A custom insert made with Superflab bolus (Mick Radio‐Nuclear Instruments, Mount Vernon, NY, USA), shown in Figure 3(c), was created to fit in the StereoPHAN (Sun Nuclear Corp, Melbourne, FL, USA) so that the Pnt‐Dos was positioned at the center of the custom insert. The StereoPHAN insert was oriented horizontally and placed at the isocenter. A dose of 250 cGy was delivered to eight different Pnt‐Dos devices from eight beam angles: 0°, 45°, 90°, 135°, 180°, 225°, 270°, and 315°. The dose measurements from these different angles were compared to the 0° readings, and correction factors were calculated.

### 2.3 Daily quality control program

A daily quality control (QC) program was developed to ensure the entire Pnt‐Dos system performed as expected before clinical implementation. The daily QC process included two steps. The first step involved performing the “Scanner Check (QA)” submodule within the Pnt‐Dos software, which performed a scanner check using 20 blank scans. This also served as a scanner warm‐up. In the second step, dedicated daily QC devices were read using a calibration curve specific to daily QC. The same set of nine Pnt‐Dos devices (0, 5, 10, 20, 40, 80, 160, 320, and 640 cGy), used for calibration in Section 2.2.1, were reused to establish a dedicated calibration curve set for daily QC, five days after the irradiation. Eight Pnt‐Dos devices that were irradiated to six dose levels served as dedicated QC devices and were read out over a three‐month period. There were one (1) device irradiated to 15 cGy, 30 cGy, 75 cGy, and 375 cGy; and two (2) devices were irradiated to 100 cGy and 400 cGy.

## 3 RESULTS

### 3.1 Calibration

The calibration curves were generated automatically using the Film Calibration submodule in the Pnt‐Dos software. Examples of such a calibration curve set is shown in Figure 2.

### 3.2 Dose accuracy

The comparison between the expected delivered doses and the reported measured doses from the Pnt‐Dos system is shown in Table 1. Because multiple readings (at least 3) are performed for each device and 15 devices are included in each dose level group, we define consistency as the standard deviation of all the readings for the same device, and its associated uncertainty as the standard deviation of the standard deviations for all devices in the same dose group. We define the measured dose as the average of the average dose readings for each device and its associated uncertainty as the standard deviation of the per‐device average readings of all devices in the same dose group. The differences between the measured and the expected doses are characterized by the absolute differences for expected doses less than 80 cGy and by percentage differences for those from 80 cGy to 400 cGy. The ranges of each metric are shown in parentheses. Dose accuracy of < ± 4 cGy for doses ≤ 80 cGy doses and < ± 3% for doses > 80 cGy are achieved on average. The maximum observed discrepancy is observed at −6.0 cGy observed for doses ≤ 80 cGy and at −5.1% for doses > 80 cGy, respectively. The 95% confidence intervals of these discrepancies based on the *t*‐distribution are (−2.3 cGy, −1.3 cGy) for doses ≤ 80 cGy and (−1.7%, −0.3%) for doses > 80 cGy, respectively. The differences between the 13000XL and V600 scanners were on average 1.3 cGy ± 2.3 cGy (−0.4 cGy, 2.6 cGy) for doses ≤ 80 cGy and 0.6% ± 2.3% (−0.9% to 2.0%) for doses > 80 cGy, respectively, with the 95% confidence intervals shown in the parentheses.

**TABLE 1:** Dose accuracy verification results for delivered doses from 15 cGy to 400 cGy, including the expected and measured doses, the consistency of the measured doses, and the difference between the measured and expected doses.

Expected dose [cGy]	Measured dose [cGy]	Consistency [cGy]	Difference (measured—expected)
15	14.3 ± 1.0 (12.3–15.7)	0.5 ± 0.3 (0.2–0.9)	−0.7 cGy (−2.7–0.7) cGy
30	28.6 ± 1.3 (26–30.5)	0.4 ± 0.2 (0.1–0.6)	−1.4 cGy (−4.0–0.5) cGy
75	71.7 ± 1.8 (69.0–74.3)	0.7 ± 0.5 (0.1–1.1)	−3.3 cGy (−6.0– −0.7) cGy
100	97.8 ± 1.9 (94.9–100.7)	1.7 ± 1.3 (0.1–3.1)	−2.2 ± 1.9% (−5.1–0.7) %
250	249.7 ± 4.6 (242.8–258.6)	3.1 ± 2.3 (0.4–6.8)	−0.1 ± 1.9% (−2.9–3.4) %
400	388.6 ± 6.3 (379.6–398.8)	5.1 ± 2.9 (1.3–9.6)	−2.9 ± 1.6% (−5.1– −0.3) %

### 3.3 Energy dependence

Table 2 shows the energy dependence results. The correction factors are the multiplicative factors needed to use the 6 MV‐based calibration curves to obtain the expected doses. These energy correction factors were derived for an expected dose of 250 cGy. An overall uncertainty of 2.3% is assigned for all energies obtained from adding in quadrature (1) the dose uncertainty of 1.9% for the 250 cGy group from Table 1, (2) 1% output uncertainty for each energy at the time of measurement, and (3) 1% uncertainty for the TG‐51 to solid water dose cross calibration.

**TABLE 2:** Energy correction factors for the Pnt‐Dos in vivo dosimetry system.

Beam Energy	6XFFF	10XFFF	15X	6E	9E	12E	16E	20E
Correction Factor	1.010 ± 0.023	1.015 ± 0.023	1.027 ± 0.023	1.049 ± 0.023	1.049 ± 0.023	1.046 ± 0.023	1.040 ± 0.023	1.021 ± 0.023

### 3.4 Angular dependence

Table 3 summarizes the results of the angular dependence measurements. The correction factors are the multiplicative factors needed to achieve the same dose as the one delivered at a gantry angle of 0°. These angular dependence correction factors were derived for an expected dose of 250 cGy. An overall uncertainty of 2.1% is assigned for all energies obtained from adding in quadrature (1) the dose uncertainty of 1.9% for the 250 cGy group from Table 1 and (2) 1% setup uncertainty.

**TABLE 3:** Angular dependence correction factors of the Pnt‐Dos in vivo dosimetry system.

Gantry Angle [degree]	45 / 315	90 / 270	135 / 225	180
Correction Factor	0.996 ± 0.021	0.984 ± 0.021	1.026 ± 0.021	1.013 ± 0.021

### 3.5 Daily QC results

**FIGURE 4:**
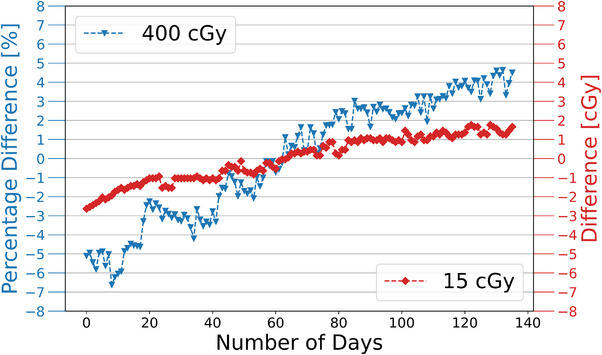
Daily QC results of the Pnt‐Dos in vivo dosimetry system over a three‐month period.

Long term stability of the Pnt‐Dos in vivo dosimetry system was monitored using the protocol described in Section 2.3 above. Figure 4 shows the absolute and percentage differences from the average reading for the 15 cGy device and one of the 400 cGy devices, as representative devices, over the whole three‐month period. Only two devices are shown for clearer presentation of the figure but the trends for the other devices are similar. Table 4 shows average day‐to‐day variations and range of variations in parentheses for all devices. Over the three‐month period, the reading increased by more than 10%, while the day‐to‐day variation was around 0.1% on average and a maximum variation of about 3%. QC devices were stored at room temperature after irradiation for consistency checks. This increase is attributed to the post‐irradiation growth of the RCF.

**TABLE 4:** Day‐to‐day variations of each Pnt‐Dos QC device.

Nominal Dose [cGy]	Day‐to‐Day variation
15	0.03 (−0.6–0.6) cGy
30	0.03 (−0.6–0.8) cGy
75	0.07 (−1.5–1.4) cGy
100 (device #1)	0.08 (−1.4–2.2) %
100 (device #2)	0.08 (−3.1–2.3) %
375	0.09 (−2.0–2.4) %
400 (device #1)	0.07 (−1.7–1.6) %
400 (device #2)	0.07 (−1.7–2.6) %

### 3.6 Initial clinical cases

Following the commissioning and characterization studies described in this work, the Pnt‐Dos system was used for 15 patients for in vivo dosimetry QA as part of the standard of care. No patient‐identifiable data were collected or analyzed for this study, and patients were not the research subjects. Most of these measurements were skin‐dose verification for electron treatments or CIED dose monitoring. To streamline the clinical workflow, we established two primary calibration sets based on 6 MV and 6 MeV beams. Since the energy dependence from 6 MeV to 16 MeV is within 1%, the 6 MeV calibration is used for all electron energies from 6 MeV to 16 MeV without additional correction. Similarly, the energy dependence of 6 MV and 6 MV FFF beams differs by less than 1%, thus the 6 MV calibration was applied to both 6 MV and 6 MV FFF beams. Additionally, because the correction factors for 15 MV and 20 MeV differed by only 0.6%, the 6 MV calibration was used for these energies with a correction factor of 1.024. Note that 10 MV FFF is not currently used in the authors’ clinic, so it was not used for patient measurements.

The Pnt‐Dos system was used in the following clinical cases: one VMAT right axilla patient (500 cGy/fraction) and five CIED patients treated with 6 MV photons; two scalp cases (200 cGy/fraction), two nasal cases (400 cGy/fraction), and one right forearm skin cancer case (200 cGy/fraction), all treated with 6 MeV or 9 MeV electrons. In all in‐field dose verification cases, the measured doses were within 5% of the expected doses. The out‐of‐field CIED cases had varying but small doses, consistent with clinical expectations. The readouts were typically performed more than 20 minutes after radiation delivery, as no calibration curve was available earlier in the reader system (see section 2.2.2).

## 4 DISCUSSION

There are few challenges to using RCF for in vivo dosimetry. First, there is a wide range of dose values that are of clinical interest in in vivo dosimetry measurements. On the low‐dose end, out‐of‐field dose measurements (e.g., to monitor delivered dose to CIEDs) are typically in the range of a few cGy. On the high‐dose end, in‐field measurements to verify prescription doses being delivered to shallow targets of some electron treatments can result in doses in the range of up to 10 Gy. The Pnt‐Dos device employs a proprietary chemical composition compared to previous variations of RCFs, aiming to provide good sensitivity and accuracy in both low and high doses.

In addition, the polymerization process continues after irradiation, resulting in continued growth of OD. In many RCF dosimetry applications, it is acceptable to wait around 24 hours or more for the OD to stabilize and minimize the systematic difference of the irradiation times between the calibration films and the application films. However, this lengthy wait time is undesirable for in vivo dosimetry applications where the results are often needed to support clinical decisions before the next fraction, which can be the next day or even later the same day. Similar to the concept demonstrated by Dunn et al for EBT3 films,^24^ the Pnt‐Dos system builds a time‐resolved set of calibration curves, which incorporates the time‐dependence of the OD growth, and it uses the appropriate calibration that corresponds to the same time‐post‐irradiation between the calibration irradiations and the patient irradiation. In addition, (RCF) dosimetry is often a multi‐step process involving careful handling of the films, resulting in cumbersome and sometimes error‐prone workflows. The Pnt‐Dos system is specifically designed for in vivo dosimetry and has streamlined the workflow by introducing automation and simplification.

In this work, we commissioned the clinical workflow of the Pnt‐Dos system for in vivo dosimetry and characterized various aspects of the Pnt‐Dos system including dose accuracy, energy dependence, and angular dependence. We also designed a daily QC program to ensure ongoing system reliability. For dose accuracy, there appeared to be a systematic, albeit small and within experimental uncertainty, underestimation on average of the measured dose compared to the expected delivered dose as shown in Table 1. This underestimation was more pronounced at some dose levels, such as 75 cGy and 400 cGy, than others, such as 250 cGy. This is possibly due to a systematic bias in the fitting function. Further investigation is underway to determine the cause. This could be mitigated by performing a separate calibration using an alternative fitting function form outside of the software module using the exported data.

The system was successfully integrated into our clinical workflow, and we have reported our initial clinical experience with its deployment.^25^, ^26^ This detector is expected to be well‐suited for TBI and TSET in vivo dosimetry. However, due to the paucity of procedures, our future work will include thorough evaluation and data accumulation.

There are a few limitations to the current work. First, the vendor requires the Pnt‐Dos devices to be kept in refrigeration at temperatures between 4 to 6 degrees Celsius.^21^ In addition, the vendor recommends removing the Pnt‐Dos devices from the refrigerator approximately 15 minutes prior to exposure.^21^ Compared to other RCFs from the same vendor, such as EBT3/4 and LD‐V1, which are stored and handled at room temperature, the special temperature requirement for the Pnt‐Dos devices may not be desirable. It is of clinical interest to explore the magnitude of errors that could be introduced when the delicate temperature control is not followed. From our clinical experience so far, minor deviations from the temperature requirements have not resulted in clinically concerning degradations of the detector system's performance. This includes storage temperature deviations of 1–2 degrees over a short period of time as well as leaving the devices at room temperature for up to 2 hours before irradiation. A systematic investigation of these effects is underway in a follow‐up study.

Second, the energy dependence of Pnt‐Dos devices was derived experimentally. It shows an appreciable magnitude of up to 5% even among the MV energies, which were greater than what studies have shown for EBT3 and EBT4 films.^27^, ^28^, ^29^ Further investigation, either thorough experiments by other institutions or Monte Carlo studies once the chemical composition of the Pnt‐Dos films becomes publicly available, is desirable to verify our findings regarding the energy dependence of the Pnt‐Dos devices.

Additionally, the calibration curves cannot be modified post‐creation in the software. However, the scanned images for all calibration scans are saved automatically. In principle, the user can develop their own workflow to process those images into calibration curves.

To further evaluate system robustness and clinical scalability, we are conducting a series of other performance tests, including inter‐scanner variability, temperature‐related differences between calibration conditions (22°C) and physiological temperature (37°C), as well as out‐of‐field measurements, such as those relevant to CIEDs. The detailed results will be reported in future, although our preliminary findings suggest that, in all evaluated scenarios, the Pnt‐Dos system demonstrated consistent and reliable performance, and no significant difference in performance was observed when the devices were used for patients compared to the in phantom studies reported here.

In addition to the vendor's Instruction for Use (IFU), the list below summarizes some lessons‐learned and recommendations from our experience:

- Ensure a stable environment for the calibration curve building process.
  - Check and disable any system updates or scheduled power‐down functions (such as those imposed by the institution) that could interrupt the building process of the calibration curve set. The length of calibration curve building is user specified. In this study, we set 24 hours as the length of each calibration.
  - Ensure stable power supply to the computer and scanner
- Read the calibration devices soon after irradiation. The time between calibration device irradiation and scanning time of the first calibration curve determines the minimum time one must wait until reading the in vivo measurement device.
- Inspect the calibration curves at all time points. The authors have observed non‐ideal fitting of the calibration curves such as spikes and overfitting. The user should inspect the calibration curves from the automated fitting procedure to ensure their integrity.
- Validate each calibration curve set before clinical use. In addition to the visual inspection of calibration curves, the user should perform independent validation of the calibration curve set by irradiating some devices to known doses and read using the established calibration set before clinical use. This validation should be performed periodically, for example, every 3–4 weeks, to confirm the validity of the calibration curve.
- Establish and validate the relevant correction factors before use, such as energy dependence and angular dependence, when appropriate.
  - Better dosimetric accuracy is expected if using a dedicated calibration curve set for the specific energy.
- Daily QC should be performed using a pair of devices, one for low dose (e.g., 15 cGy) and one for high dose (e.g., 400 cGy)
- Perform the scanner warmup before daily QC or reading patient's in vivo device when the scanner has been idle for a significant amount of time (e.g., > 8 hours).
- The device holder for the XL series of scanners is designed to be slightly smaller than the scanner's glass window. Therefore, it is recommended that the user establish and mark the device holder position on the scanner, so the auto‐generated ROIs are in the desired locations. Separate calibration curves should be established for each scanner when multiple scanners are used in a clinic.
- The QR code may sometimes not be recognized by the software during scanner such as due to damage to the QR code or manufacturing deficits. When this happens, there will be no auto‐detected ROI generated. The user can reposition the device within the slot with a slight wiggling motion and repeat. If it is still not recognized, one can place another dummy device in an empty slot within the device holder and move the auto‐detected ROI of the dummy device to the device of interest for dose reading.
- Use an unexposed device when reading devices with low expected doses. A reference device is typically used in dose readings. This can cause darkening of the reference devices due to the prolonged exposure to ambient temperature as compared to those unexposed devices stored in the refrigerator. If this type of reference devices is used to read a patient device with low expected doses, such as in measurements of out‐of‐field CIED devices, the software will report negative readings due to the subtraction of the reference device reading from the raw low dose patient device reading. This can be mitigated by reading an unexposed device taken directly from the refrigerator, instead of a reference device, and subtract its dose reading from the patient device dose reading instead. The software will warn the user about the absence of the reference device but will not prevent the user from reading the devices.
- To assess the usability of an expired lot, the device can be re‐commissioned to generate another set of calibration curves. Calibration is then validated by irradiating two new devices at high and low doses. This procedure can be repeated every 3–4 weeks or as frequently as the physicist deems appropriate.

## 5 CONCLUSIONS

A new (RCF)‐based in vivo dosimetry system (Pnt‐Dos) was successfully commissioned for clinical implementation. The system demonstrated a dose accuracy within ± 5 cGy for doses ≤ 80 cGy doses and within ± 5% for doses > 80 cGy, with an observed energy dependence of up to 5% across commonly used MV photon and electron beam energies. These results confirm the system's suitability for clinical use, provided that consistent QA and routine monitoring are maintained. Although the system offers flexibility and ease of use, adherence to established (RCF) dosimetry protocols remains essential for achieving accurate and reproducible results. Finally, we hope this work could serve as a practical reference for future clinical users and facilitate broader adoption of film‐based in vivo dosimetry in radiation therapy.

## AUTHOR CONTRIBUTIONS

All authors contributed to the conception and design, acquisition of data, analysis and interpretation of data, and drafting and revision of the manuscript. All authors reviewed and approved the final version of the manuscript.

## CONFLICT OF INTEREST STATEMENT

Maria Chan has a research grant from Ashland Inc., the manufacturer of the Gafchromic films. Other coauthors have no conflict of interest to disclose.

## Data Availability

Authors will share data upon request to the corresponding author.
